# Employee wellbeing and cost reduction drivers of corporate social responsibility: Evidence from Congolese mining sector

**DOI:** 10.3389/fpsyg.2022.850283

**Published:** 2023-02-06

**Authors:** Huaping Sun, Grace Mulindwa Bahizire

**Affiliations:** School of Finance and Economics, Jiangsu University, Zhenjiang, China

**Keywords:** corporate social responsibility, employee wellbeing, cost reduction, mining sector, CSR implementation, lack of resources

## Abstract

This research proposes an internal organizational framework that explains why firms implement corporate social responsibility (CSR). Based on the perspective of managers in the mining sector of the Democratic Republic of Congo (DRC), wellbeing of employees and cost reduction were identified as internal driving factors of CSR. This study was carried out on a sample of 103 mining, using a structural equation through LISREL. The findings of the study reveal; first, that firms' pursuit of cost reduction positively influences the implementation of CSR; second, firms are driven by CSR in order to ensure the wellbeing of their employees, fostered by compliance with labor standards in the mining sector. Therefore, CSR compliance was included as a mediating variable. Lastly, the lack of resources remains a barrier to CSR implementation. This research adds to the growing body of literature on CSR antecedents by demonstrating that in the Congolese mining sector, CSR implementation is linked to the wellbeing of employees as well as compliance to labor standards and regulations, cost reduction, and financial resources as well as human resources. This research responds to deficiency in novelty and lack of academic studies on drivers of CSR in the mining sector in Sub-Saharan regions.

## Introduction

The concept of corporate social responsibility (CSR) has several definitions, not clear boundaries, and has been part of the academic and business vocabularies for decades (Dahlsrud, 2008). CSR has diverse potential benefits, such as economic performance, reducing environmental impacts and ensuring the wellbeing of employees. CSR also allows for firms to improve their reputation and competitive advantage (Eyasu and Arefayne, 2020; Maráková et al., 2021).

Due to recent ethical, social, and environmental scandals caused by large companies around the globe, companies are increasingly engaging in social responsibility. However, the question that is posed is whether the attention to CSR was born out of an ethical approach that tries to improve the social context and preserve the environment, or rather of a pressure that weighs on the company and dictates its strategic choices. Conventionally, the primary objective of business was to maximize profits (Tuyen, 2015). Nowadays, firms seem to have various objectives fixed by managers, servile objectives rather than profits, while considering long-term rather than short-term profit, compensation for shareholders, brand image, business growth, and respect for the environment. Majority of recent research is examining the motivations of firms to implement CSR, and it is found that some of the motivations are linked to disciplines such as marketing, human resource management, and financial performance, and that the other motivations are more specific such as competitive advantage, and fear of sanctions due to non-compliance with regulations (Viswanathan, 2017; Latapí Agudelo et al., 2020; Luisa and Alwyia, 2020).

For instance, the mining sector also joins in several CSR initiatives more than any other sector. On the other hand, this can be explained, but mounting pressures from various external stakeholders such as local communities and NGOS, which have been a push factor for implementing CSR in mining firms.

For instance, a series of reports has shown that mining in the DR Congo is plagued by problems like human rights abuses and unsafe mining, and several other risks have been reported in the Congo mining industry. Amnesty International reported that in 1 year 72 artisanal cobalt miners died because of tunnels that collapsed and other underground incidents (Sovacool, 2021). Such situations require firms in this sector to consider CSR by also adopting new environmentally friendly technologies for sustainable processes and outcomes. There is a remarkable lack of academic studies on driving factors for implementing CSR in the mining sector in Sub-Saharan Africa. It has been noticed that most studies on CSR in this region chose other industries (Mzembe et al., 2016). Most studies are concerned with other specific CSR-related problems rather than motivations that push firms to integrate CSR (Bannister and Brynne, 2008; Calvão et al., 2021). While we did not find any study on CSR drivers in the mining sector of the Democratic Republic of Congo, a similar remark was made earlier in Malawi, where the only research of Mzembe and Meaton (2014) among the rare studies attempted to study the drivers of CSR in the mining sector in Malawi by conducting a case study on a single company. Their study revealed a significant influence of external factors on CSR implementation through stakeholders ‘perspectives. There is still a big gap in the existing literature on the sub-Saharan region relative to CSR drivers in the mining sector; there is also a need to identify the influence of the internal drivers on the implementation of CSR in this sector. On a very positive note, new perspectives could be added to the literature in this region by surveying several companies rather than one single case. In least developed countries, for instance, firms are still questioning the benefits of integrating CSR (Elifneh, 2020). The perspective of most researchers holds that managers might give CSR more attention if they could expect CSR actions to help them bridge the gap between revenues and implied costs of CSR actions. Internal drivers could be profit- oriented, as managers always look at the lens of economic returns before accepting any cost imposed by CSR initiatives. Thus, firms are committed to CSR to create a good impression, to attract customers (Luisa and Alwyia, 2020), increase their competitive advantage (Eyasu and Arefayne, 2020), and solve their previous mistakes using their CSR initiatives (Brandenburg, 2013). The aim of this study is not entirely in contrast with a profit-oriented perspective. However, we look at a different angle where the internal mechanism supports the momentum of business in the long run in a sense that CSR practices can be taken without altering business profit and market share.

For instance, the wellbeing of employees in other sectors in the western region has been a serious concern for CSR. Firms implement CSR in order to guarantee the wellbeing of employees; Fukukawa and Moon (2004) illustrated in an empirical study that minimizing the risk of accidents was the main cause of implementation of CSR approaches by Japanese companies. In the extractive and mining sectors for instance, some criticisms led to implementation of measures and initiatives that will ensure the wellbeing of workers. In this regard, it appears that CSR implementation responds to the emergency of enhancing the wellbeing of workers.

Several studies have looked at the benefits of CSR relative to the practice of human resource management, where CSR appears to be an HR tool that could be of help in various facets such as employee retention, employee performance, organizational commitment, and employee satisfaction (Hofman and Newman, 2014; Barakat et al., 2016; Chaudhary, 2018; Fanglin et al., 2019). Overall, good HR CSR seeks to guarantee the wellbeing of employees. However, this could also be looked at from a different perspective, as for instance researchers sought to identify the drivers that lead employees to participate in CSR activities, and at a different angle researchers clearly could also assess the wellbeing of employee as the predictor of CSR implementation. Although only few studies have attempted this, this study goes beyond the voluntarily aspect of CSR by drawing the attention toward the role of compliance to labor standards.

Another internal aspect is firms are much concerned on the gap between cost of CSR initiative and economic return. However, in the long run, CSR-related practices and initiatives through green technology will help in reducing operating costs in production to some extent (González-Benito, 2005). Thus, few studies confirmed that firms are motivated by reduction of loads and production costs to implement CSR (van Rekom et al., 2013). However, due to the particularity and nature of operations in each sector, this remains unclear among firms in the mining sector, as various sectors may reflect distinctive perspectives on the relationship of cost reduction and CSR implementation. Therefore, there is a need to look into the extractive and mining industries.

Although it can be assumed that the two motivations mentioned above drive firms to integrate CSR, lack of resources could still be a hindrance despite the internally driven motivation to implement CSR. Litvinenko, Tsvetkov, and Molodtsov (Litvinenko et al., 2020) argued that lack of resources could negatively affect organizations in effectively implementing CSR. Although firms show clear intentions in integrating CSR initiatives, the lack of relevant resources becomes a considerable barrier to CSR implementation. Porter and Linde (1999) argued that CSR is a competitive instrument that needs necessary resources. The research of Castka et al. (2004) put it this way: lack of information at the company level on specific clean technologies adds to risks and uncertainties with regard to the adoption of CSR.

In this study, the researcher seeks to explain the internally driven motivation for CSR implementation in the mining sector of the DRC, a sub-Saharan region. This study seeks to address the limitations of previous studies on this region by revealing factors that are rarely studied.

## Literature review

The first stage in implementing CSR, according to various academics, is for firms to understand the motivation and obstacles in putting CSR initiatives in place; therefore, internal and external motivations and barriers should be identified (Al-Abdin et al., 2018; Alotaibi et al., 2019). However, firms employ CSR practices through the instrumental and normative approaches according to Berman et al. (1999). The normative approach claims that businesses have a moral obligation to address the interests of their stakeholders, whereas the instrumental approach claims that stakeholders believe CSR initiatives can improve a company's financial success. The complexity of motivating factors and barriers that affect CSR was recognized by academics, industry participants, and other interested parties (Fabrizi et al., 2014).

Several research studies have looked into the motivation that firms have toward CSR implementation, such as Lu et al. (2020), who empirically illustrated that firms adopt CSR as a result of political relationship, institutional environment, executive characteristics, and customer expectations, whereas Yin (2017) earlier argued that factors such as ethical corporate culture and management commitment are defined as internal factors, and that globalization problems, political issues, and normative social pressures, are classed as external factors. These factors have an impact on how organizations operate when it comes to implementing social practices.

In a research study on Spanish companies, Agudo-Valiente et al. (2017) established that barriers and drivers of CSR may be categorized into those that are impacted by managers' moral convictions and those that are not; they are then categorized as subjective and objective. Managers frequently cite stakeholder pressure, institutional framework, and reputation management as objective drivers, while subjective drivers include incorporation of sustainable development concepts and ethical integration into daily operations. Managers consider philanthropy, charity, and public relations as subjective motivation, and objective CSR barriers include lack of financial and human resources, people, and time to adopt CSR initiatives.

Siria et al. (2017) established that firms engage in CSR activities in order to gain and create a solid reputation, acquire new sets of costumers, recruit and retain the best staff, and promote innovation (products, process, environmental and social innovations, etc.). The most common driving motivations for implementing CSR activities, according to the European Survey on CSR, were ethical/moral commitment, top management priority, risk management, and market positioning. By comprehensive literature research Alotaibi et al. (2019) identified seven barriers to CSR in KSA where experts were interviewed. Shen et al. (2015) identified 12 barriers to CSR in their extensive literature review: lack of stakeholder awareness, lack of training, lack of information, lack of financial resources, lack of customer awareness, lack of reputation value, lack of knowledge, lack of regulations and standards, diversity, company culture, lack of social audit, and lack of top management commitment. Financial restrictions are the most significant hurdle to CSR implementation in the Indian textile industry according to the authors, followed by customer awareness, lack of norms and standards, and lack of top management support. For instance; nowadays consumers are increasingly concerned about the social responsibility of firms. Furthermore, firms integrate CSR to create for themselves opportunities, because CSR is seen as a source of competitive advantage (Maráková et al., 2021), and it facilitates, to some extent, access to the international market.

Stojanović et al. (2020) established a framework that explains CSR implementation. In doing so, they underlined the aim of implementation, wherein CSR activities were considered as the variables that impact employee loyalty in addition to the firm's performance.

In general, only a few research have looked at employee wellbeing as a driving factor of CSR, although studies such as Bansal and et Roth (2000), Barnett and Salomon (2006), Chan and Wong (2006), Egels-Zandén (2009), and Thien (2011) earlier attempted to investigate cost reduction in production as well as employee wellbeing as driving factors toward the implementation of CSR. However, this research is focused on the mining sector and goes further by investigating lack of resources as a barrier to CSR implementation in this sector.

### CSR implementation

Among the various definitions attributed to CSR, this concept has been frequently associated to four dimensions, economic, legal, ethical, and philanthropic. Moreover, some researchers expand it up to five dimensions, economic, stakeholder, environment, social, and voluntariness (Dahlsrud, 2008). Emphasizing on the last dimension (Turker, 2009), CSR goes beyond simple compliance with rules and regulations. In other words, integrating CSR is only not limited to complying with socio-environmental regulations. CSR implementation is described as the extent of management efforts to do more than regulatory compliance and to ensure socially and environmentally sound practices are taking place in the work environment. This is an important element of CSR because of the need for the top management to ensure that the right thing is being done, and can be translated as the driving force of CSR (Moomen et al., 2019).

Despite the fact that adoption and implementation are major topics in international business literature and the fact that the two notions have been used interchangeably in the literature, they can be distinguished per definition. Indeed, the current literature frequently interchanges the terms “adoption” and “implementation,” The two concepts as “externally” and “internally” oriented practices: “adoption is primarily a response to external pressures for change that signals compliance but may not alter operations while implementation is indicative of organizational commitment (Risi, 2016). Adoption might thus occur for external reasons without being translated into the firm's operations, whereas implementation necessitates a financial investment in the new practice. This definition establishes a useful contrast between the two notions, but it does not eliminate them.

Typically, CSR effectiveness in firms means that firms evaluate and consider the impact of their practices on the environment and social wellbeing as their responsibility. More precisely, the efforts of firms on CSR activities should be far above the expectations of regulatory or environmental institutions. However, CSR does not necessarily mean that firms are giving cash to assist in term of charities or are involved in a kind of philanthropic initiatives, although it is part of it. Effective CSR starts when a firm recognizes the environmental, economic, and social consequences of running its business and initiates programs to correct or address them (Goyal, 2006; Massarani et al., 2007), whereas CSR compliance is the extent to which firm complies with labor regulations and laws (Newman et al., 2018). CSR activities in the mining sector have been defined as the combination of legal compliance and philanthropic dimension, and this definition is aligned with various definitions of CSR commonly used in previous studies.

### Cost reduction

Earlier it was perceived as a barrier to corporate profitability, and adopting CSR is not always synonymous with colossal investment with profits. Porter's hypothesis refuted this traditional economic view, which stems from the research study of Porter and Van der Linde (1995) who stressed that integration of environmental approaches does not necessarily cause a drop in profits and can, on the contrary, stimulate a firm to shape its strategy by combining green innovation and economic benefits, which is a “win-win” strategy. The reduction of production costs properly exemplifies the “win-win” strategy, as it is defined as the reason for enterprises to integrate CSR in order to cut their production costs and boost profitability. In this logic, the integration of CSR through practices such as installation of clean technologies or recovery and recycling of waste makes companies save raw materials and energy, which would reduce their production costs. In this case, firms tend to set up management practices, environmental factors, which help in cost reduction and profits maximization (Quazi et al., 2001; Céspedes-Lorente et al., 2003; González-Benito, 2005; Melsa, 2006). Similarly, Shrivastava (1995) argued that organizations have the potential to lower operating costs by harnessing ecological efficiency.

Margolis et al. (2009) also suggested this in a meta-analysis where they found that company environmental policies were positively related to profitability. Likewise, Melnyk et al. (2002) stated that cost reduction and better product quality seemed to be the principal outcome for the implementation of CSR through a study on more than 1,500 organizations in America. The Canadian Affairs for Social Responsibility report (CBSR, 2003), on the involvement in CSR by Canadian SMEs, highlighted that CSR in Canadians SMEs is closely linked to reducing costs, increasing economic performance, and financially viable situations, thanks to more eco-efficient management. However, this has severally been empirically studied, for instance the Porter's hypothesis still seems mixed. Research studies that jointly considered various levels of CSR (environment, human resources, community activities, etc.) contradicted the conclusions of the studies cited above (Barnett, 2007). More directly, other researchers like Cerin (2006) went so far as to strongly question the theoretical foundations of Porter's “win-win” hypothesis.

### Employee wellbeing

Employee wellbeing in this context refers to occupational health and safety that firms should implement for the welfare of their workers (Wright, 2000; Montero et al., 2009; Kara et al., 2013). For instance, issues related to human rights and child labor have been catching the attention of various activist as well as researches. Nowadays, businesses have become more conscious on enhancing the wellbeing of workers (Montero et al., 2009; Leon-Kabamba et al., 2018). Thus, the wellbeing of workers has a significant connection with the performance of employees at work (Chaudhary, 2018), whereas the wellbeing of employees in this sector is a challenge (Calvão et al., 2021) and a serious question that raises a huge attention of NGOs and researchers. CSR is expected to start in the workplace where workers should achieve their tasks in an environment that responds to standards (Barakat et al., 2016). Thus, it can be assumed that the implementation of CSR in firms is motivated by the need to ensure the wellbeing of workers, especially when it has to do with their health and safety in the workplace.

### Research hypotheses

**Figure 1:**
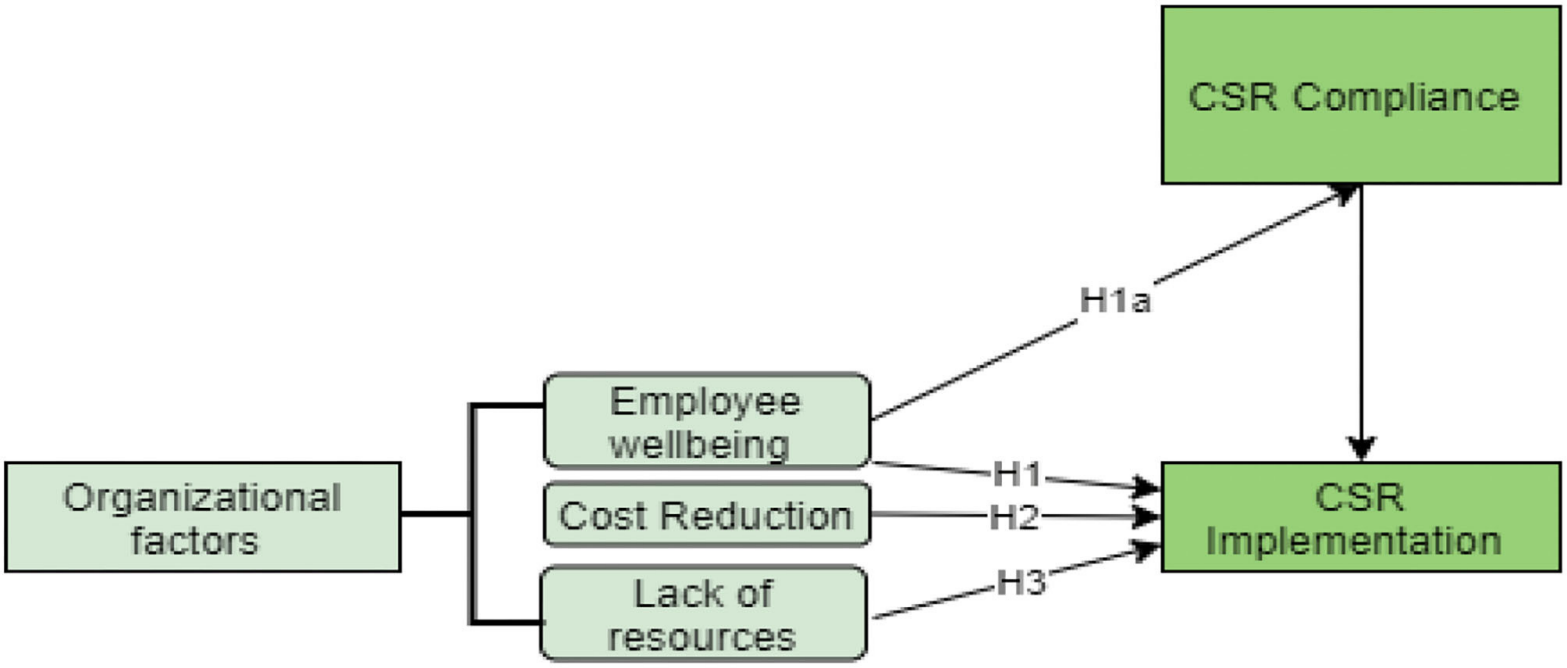
Conceptual framework.

This study proposes a conceptual model of the research that explores a large and very heterogeneous body of literature on motivations toward CSR implementation, and several studies proposed multiple reasons that push organizations to implement CSR policies. However, based on the various motivational factors that are mentioned in this study, this research suggests a number of hypotheses in order to address the assumptions on motivations for effective CSR implementation in the mining sector. Figure 1 shows the conceptual framework of the study.

#### Employee wellbeing and CSR implementation

Employee wellbeing is considered as motivation to engage in CSR; therefore, it is defined as a factor that drives firms to get involved in CSR while aiming to improve the wellbeing of employees in areas such health and safety of workers in the workplace. Thus, improvement in employee wellbeing, in particular health and safety at work, is recognized by some researchers as the primary motivation for firms to engage in CSR initiatives, whereas Boiral (2006) stated that industrial pollution is a matter of health and safety of workers in a factory before it becomes a serious environmental problem. In fact, workers appear to be the first victims of pollution, especially in a sector such as mining where workers are highly exposed since they are generally found as close as possible to the production, which is the source of polluting discharges.

In this sense, Fukukawa and Moon have shown that reducing the risk of accidents to a minimum was the main cause of the implementation of CSR approaches by Japanese companies. With this in mind, Werner (2009) estimated that traditional CSR activities encompass “health, education, and community services that are generally seen as distinct and unrelated to basic business operations.” Commitment to CSR in order to improve health and safety conditions can be claimed by employees through the use of internal intermediaries such as hygiene committees or environment departments. Likewise, many empirical studies have established a positive relationship between engagement in CSR and improved wellbeing, i.e., the study conducted by Quazi et al. (2001) who asserted that companies engage in environment processes in order to ensure the wellbeing of employees. Hence, most managers must have realized that implementing CSR initiatives partly influences the productivity of their workers; for instance, on the issue of mining firms, the wellbeing of workers is very crucial.

Consequently, the existence of a relationship between improvement in the wellbeing of employees, in particular health and safety at work, and commitment to CSR may explain the increased intensity of companies' commitments in CSR. This observation leads us to the following hypotheses, which are:

H1: The wellbeing of employees positively influences the effectiveness of CSR implementation.
H1a: CSR compliance partially mediates the relationship between wellbeing of employees and CSR implementation.

#### Cost reduction by firm's effective CSR implementation

Nevertheless, several researchers have continued to demonstrate the positive impact of CSR on savings, following the integration of CSR, (Bansal and et Roth, 2000; Egels-Zandén, 2009; Margolis et al., 2009; Campopiano et al., 2012). Chan and Wong (2006) clarified with their research in that the implementation of CSR could reduce the energy consumption, water, and other raw materials, an evidence of hotel industry. Likewise, Campopiano et al. (2012) asserted that the mainstreaming of CSR has been driven by cost savings directly, as it leads to improved financial portfolios. The majority of the literature seems to affirm that the voluntary engagement of companies in CSR is motivated by reducing production costs and costs. This leads us to the hypothesis which concerns the organizational level:

H2: Reducing production costs positively influences he effectiveness of CSR implementation

#### Lack of resource and CSR implementation

Lack of resources is defined in this research as the financial and human resources required by the social and environmental approach that slow down the motivation of companies to engage in CSR. This motivation is mainly made up of three types of barriers that can slow down the firm's involvement in CSR. First of all, the lack of financial means despite the potential for a production that is more respectful of the environment and generates substantial savings and costs.

The integration of social responsibility in a company can be inhibited by several financial obstacles. First of all, costs of implementing CSR practices present an obstacle to the integration of CSR, especially in SMEs. The report of Canadian Affairs for Social Responsibility (CBSR, 2003) asserted that industrial regulations and costs of clean technology prohibit SMEs from integrating CSR. In addition, there is little government aid and internal investment policies are incompatible with CSR objectives. Then, in a broad sense, the lack of human resources (qualified personnel) and different aspects of the work, of a company can present obstacles in the integration of CSR. In this sense, problems can arise when, there is lack of appropriate skills that prevents companies from engaging in CSR (Carlisle and Faulkner, 2004; Sweeney, 2007).

Finally, lack of information is a serious obstacle in the integration of CSR, and can be translated to inaccessibility of information and appropriate expertise. Despite the significant potential of CSR to improve the competitiveness of companies, in many cases, companies are unable to exploit these opportunities because of ignorance. Lack of information at the company level on specific clean technologies, thus, contributes to risks and uncertainties with regard to the adoption of CSR, (Castka et al., 2004). This lack of information is observed in particular at the level of SMEs and often prevents them from integrating CSR (DeTienne and Lewis, 2005). Empirical studies have confirmed these findings (Arevalo and Aravind, 2011; Laudal, 2011), like the study carried out by Santos (Santos), which has shown that lack of resources can slow down the commitment of companies in voluntary environmental initiatives.

Along the same lines (Laudal, 2011), Arevalo and Aravind have shown that lack of resources negatively influences the commitment of companies to CSR. Internally, lack of financial and human resources has often handicapped companies in their strategic approaches. In addition, lack of resources slows down companies' commitment to CSR. This brings us back to the hypothesis that is:

H3: Lack of resources (financial and human) negatively influences the effectiveness of CSR implementation

## Research methodology

### Context of the research

The research population of this study consists of executives from all registered mining firms that are operating in the Democratic Republic of Congo. As per definition, a research population is a collection of individuals who share similar characteristics based on what a researcher is interested in and therefore qualify to be included in the study (Gravetter and Forzano, 2018). Within the context of research, a population is defined as a group of individuals taken from the general population who share common characteristics such as age, sex, and work conditions who are researched on because of their relevance to a research question (Bryman and Bell, 2015). Mugenda and Mugenda (2003) suggested that in explanatory studies, 10% of the survey population is representative enough to generalize the characteristics being observed.

Mining activities in the DRC are regulated by Law in the Mining Code and Decree No.038/2003 of March 26, 2003 laying down relevant implementing measures that firms ought to follow. Furthermore, beyond the Mining Code and the Mining Regulations which establish their implementing measures, other legal and regulatory texts contain provisions related to the mining sector that mining companies are expected to comply with. The mining code calls for mining companies to comply with regulations. Hence, in this, an entity is set up for inspection and control of mining and quarrying activities in terms of safety, hygiene, work management, production, and transport, marketing and social. This entity is also responsible for compilation and publication of statistics and information on the production and marketing of mining and quarrying products. Companies are expected to comply with these basic regulations.

### Sample

In this research, cluster sampling was conducted to group mining firms into different clusters based on geographical location. Then, the convenient sampling technique was used to select specifically mining firms operating in different city of DRC. The criteria for selection were: the selected respondent should be an executive of a firm with requisite information about the operations of the firm. The selected executives included operational managers and HR managers who are responsible for day-to-day management of the firms and are able to provide adequate information on the operations of the firms.

This research is established on a sample of 103 firms in the mining sector of the democratic Republic of Congo. A total of 200 firms were reached out to participate; however, only 103 responded. The response rate of 51.5 % is acceptable, referring to previous studies (Krishnan and Poulose, 2016) that considered 50% and above as an acceptable response rate to proceed with analysis while taking into account the limitations faced in this particular field of investigation. However, this rate is moderately fair but not ideal. This response rate can be justified by the lack of willingness of the companies contacted and bureaucracy in the Congolese administration; these added to the problem of poor development of information and communication technology at the level of mining companies in DRC.

### Measurement

#### Cost reduction

A number of studies have used the variable “cost reduction” to determine what motivates companies to implement CSR with the aim of reducing their production. This variable is coded cost_red. In a United Nations report coordinated by (G, 2008) on a study that addresses CSR in Caribbean countries, the researchers used a set of items concerning production cost in order to measure the motivation of 365 Caribbean companies to engage in CSR to reduce their costs of production.

In the same way, (AL) in an exploratory study on the motivation commitment of companies to CSR measured the variable “reduction of production” with a unit of items used to interview 48 Australian companies. Among the many studies that also used the variable cost reduction of production, we found the study by Chan and Wong (2006). By analyzing the empirical studies available in the literature on motivations for engaging in CSR by reducing production costs, we identified three subjects that come up quite often in the studies: energy consumption, operational risk, and profitability.

Thus, we targeted our questions on these three subjects to have, on the one hand, very accurate answers on the impact of the variable used, and, on the other hand, we chose to stay on the three questions so as not to have a long questionnaire that reduces our chances of having answers.

#### Employee wellbeing

The employee wellbeing variable measures the intensity of the company to engage in CSR in order to improve the wellbeing of employees in the area of health and safety in the workplace. This variable is coded emp_welb. Several studies have considered wellbeing of employees as a variable that explains the commitment of companies to CSR. Thus, with the aim of operationalizing the variable “employee wellbeing,” a United Nations study (G, 2008) used four items to question 365 Caribbean companies on the impact of the influence of this variable as well as to CSR. Similarly, in an empirical study that sought to establish a relationship between wellbeing of employees and motivation to engage in CSR, Legendre (2008) posed two questions on 48 Australian companies to verify the existence of this relationship (Legendre, 2008). Another group of researchers (Quazi et al., 2001) used four items to verify the hypothesis that suggests that the intention to comply with the ISO 14000 standard is motivated by the need to ensure the wellbeing of employees. On the other hand, Tanur and Jordan (1995) and Llorente and Macia (2005) used two items. The overall satisfaction index and the index of the quality of life at work constructed from several items in order to measure employee satisfaction at work.

#### Measures for lack of resources

This variable measures the intensity of financial and human resources required by the social and environmental approaches that slow down the motivation of companies to engage in CSR. This variable is coded as “la_resou”; in terms of the literature, there is no scale used to measure this variable. Nonetheless, various researchers have attempted to measure this variable using the example of the study carried out by Santos (2011) on 2000 Portuguese small and medium-sized enterprises; the latter used 6 items in order to identify barriers that hinder companies in the process commitment to CSR. For his part, Legendre (2008) used a series of 12 items on a Likert scale of 4 levels that goes from 1 (it is not an obstacle) to 4 (it is a major obstacle) in order to measure the impact of obstacles on motivation for integrating CSR. In the same sense, Arevalo and Aravind (2011) used 5 items that revolve around three questions: lack of financial resources, lack of human resources, and lack of time to devote to CSR. These items are measured on a 6-level Likert scale. Based on the definition of the variable “lack of resources” and the studies cited above, we have distinguished four subjects that always come up in questionnaires on barriers or brakes, namely, lack of financial means, human resources, skills, and time.

#### CSR implementation

This variable measures the extent to which firms are implementing CSR initiatives and programs, and it can also be defined as management of CSR activities in firms; this variable is coded CSR_MGT. Few studies have used the variable CSR, but in most of them, it was operationalized by five measurement items. Schreck (2013) and Shin and Thai (2014); whereas Newman et al. (2018) used four items that explain the extent to which firms have a welldeveloped CSR strategy at the management level that goes beyond compliance with existing regulations.

## Data analysis and results

In this study, we relied on structural equation modeling, commonly known as SEM, as it is welldefined by its uniqueness of having various multivariate techniques integrated in a single model framework; it includes factor analysis for latent variables, and it gives the possibility to conduct path analysis or regressions modeling.

### Confirmatory factor analysis

**Figure 2:**
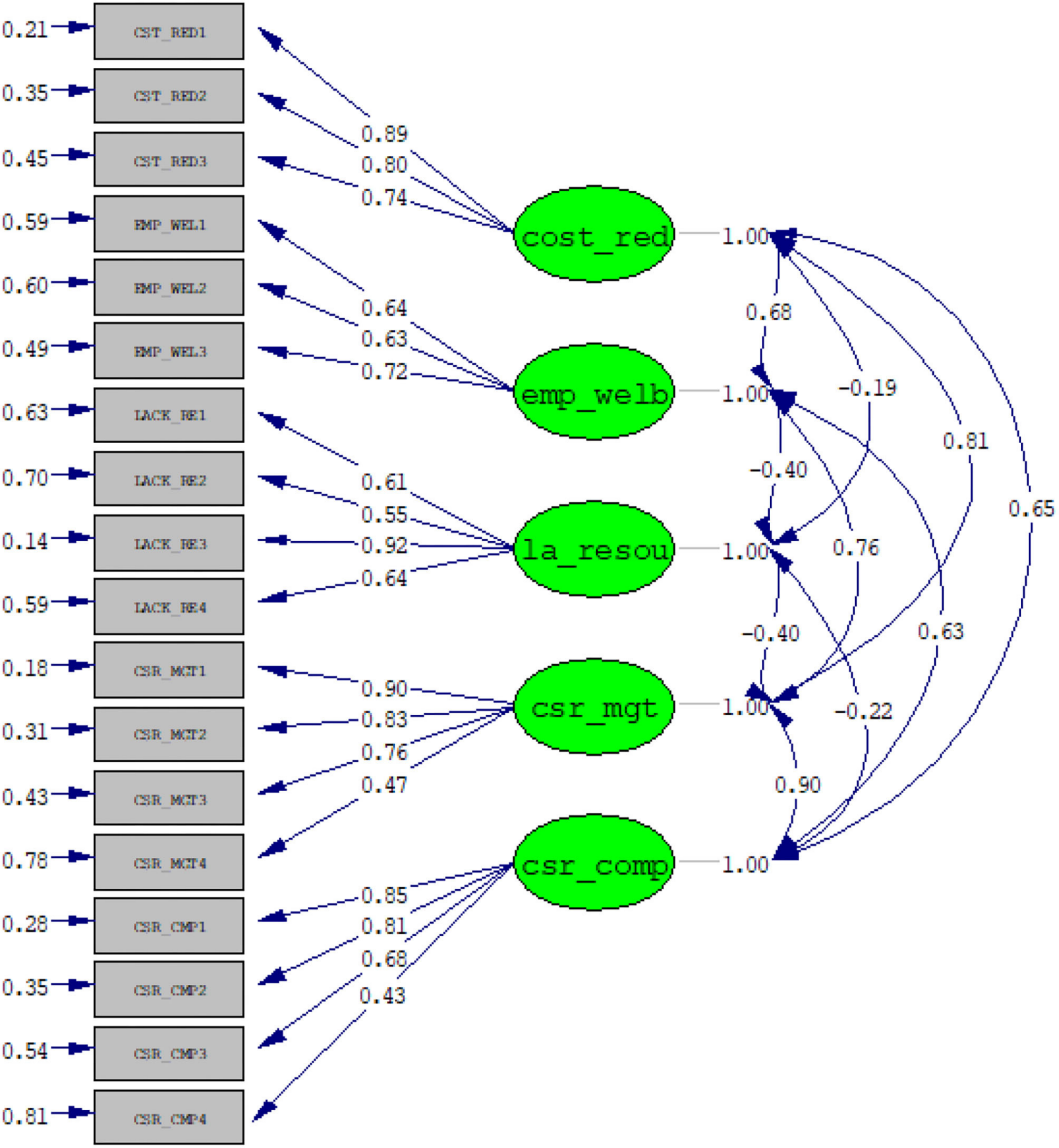
Confirmatory factor analysis.

Figure 2 illustrates the presence of significant correlations among the constructs; additionally, the measuring items show high loadings to the specific constructs. Therefore, looking at the paths in the above figure, it clearly depicts, that the internal factors are highly correlated with the indicators of corporate social responsibility of this study (CSR compliance, CSR implementation), The path from Cost reduction (cost_red) to CSR implementation (csr-mgt) demonstrates strong correlation (β = 0.81), and Cost reduction with CSR compliance β = 0.65. The construct employee wellbeing (emp_welb) also shows a strong positive correlation with both CSR indicators,; the correlation between employee wellbeing (emp_welb) and CSR implementation is β = 0.76 and with CSR compliance is β = 0.63. The correlation between lack of resources (la_resou) and CSR compliance is negative (β = −0.4); furthermore, lack of resource is negatively correlated with CSR compliance (β = −22). The CSR indicators are strongly correlated; as can be seen in the path between CSR implementation (csr_mgt) and CSR compliance (csr_comp), the correlation is positively significant (β = 0.9).

Looking at the inter-correlations among the constructs, it can also be concluded that the perceptions that firms have in achieving lower production is positively related to their CSR implementation. Moreover, that fact of being motivated to guarantee the wellbeing of employees pushes firms to comply with CSR and enforce labor standards in their day-to-day operations. However, resources seem to be a key element, as based on the perception of the managers lack of resources prevents firms from complying and enforcing CSR, the reason why lack of resource is negatively correlated to CSR effectiveness. Therefore, implying and balancing the three internal factors effectively, firms are likely to be effective and efficient in the formulation and implementation of their CSR strategy. However, as the variables of internal motivations were not used separately from each other, they demonstrate a significant relationship among them, although it can be noted that all the variables were negatively correlated to lack of resource (la_resou).

Composite reliability (CR) and average variance extracted (AVE) were also obtained, as can be seen in Table 1, through the results of the model by confirmatory factor analysis (CFA). Therefore, we were able to obtain satisfactory values from the standardized factor loading of CR and AVE for different measurement items for different constructs that are presented in this study. Table 2 indicates the goodness fit of the model.

**Table 1:** Confirmatory analysis results.

**Latent variables**	**Observed variables**	**Factor loading**	**CR**	**AVE**
COST REDUCTION (cost_red)	Cost_redu1	0.89	0.853	0.66
	Cost_redu2	0.8		
	Cost_redu3	0.74		
EMPLOYEE WELLBEING (emp_welb)	emp_welb1	0.64	0.7	0.5
	emp_welb2	0.63		
	emp_welb3	0.72		
LACK OF RESOURCES (la_resou)	La_resou1	0.61	0.8	0.5
	La_resou2	0.55		
	La_resou3	0.92		
	La_resou4	0.64		
CSR implementation (csr_mgt)	csr_mgt1	0.9	0.837	0.6
	csr_mgt2	0.83		
	csr_mgt3	0.76		
	csr_mgt4	0.47		
CSR compliance (csr_comp)	csr_cmp1	0.85	0.8	0.5
	csr_cmp2	0.81		
	csr_cmp3	0.68		
	csr_cmp4	0.43		

**Table 2:** Model fit.

**Model fit**	
Chi-square x^2^	345
degrees of freedom DF	128
Chi-Square divided by DF	2.6
RMSEA	0.07
Tucker-Lewis Index (TLI)	0.91

The chi-square of the measurement model is 345, with a *p* < 0.05 and showing a significant difference between the latent variable and the observed one. The degree of freedom is 128, which is considerably large; moreover it is also needed to verify the value of the chi-square divided by the degree of freedom. Chi-square divided by DF is equal to 2.6, which responds to the test rule, where it has been suggested that the threshold is × 2 ≤ 5. Therefore, other indices were also used to assess the fit of the model such as RMSEA (0.07), 90% confidence interval (0.03 to 0.09), and Tucker-Lewis Index (TLI, 0.91), which were satisfactory in assessing the fit of the measurement model; the indices are within the recommended threshold, which indicates an adequate fit of the model. As a result, the measurement model was accepted.

In this study, the researchers carried out validity and reliability analyses and factor analysis, (as illustrated in Table 1). Most items presented values that exceeded the threshold of 0.7, which is satisfactory when referring to (Cronbach, 1951). The internal consistency and reliability respond to the test rule, because all the values exceeded 0.7. However, the convergent validity test rule suggests that the average variance extracted (AVE) estimates have to exceed 0.5 (Bagozzi et al., 1981). In this study, the results respond to this test rule, as can be seen in Table 1; for all the variables, the value of AVE is >0.5. The data of this study present satisfactory internal consistency and convergent validity that allowed for carrying out the investigation. Moreover, for composite reliability (CR), Bagozzi et al. (1981) suggested that the CR value should be more than 0.7. In this study the CR values for all the constructs range between 0.7 and 0.85. Therefore, the study presents satisfactory reliable numbers.

In Table 1, the statistics are necessary for the evaluation of discriminant validity. Firs, in previous analyses, it was found that there was convergent validity since the average variance extracted (AVE) for each factor is 0.5 and above. Additionally, construct reliability, which is another measure of convergent validity, is also >0.7, indicating high internal consistency. According to the recommendations of Bagozzi et al. (1981), the CR and AVE values are all satisfactory.

### Discriminant validity

Finally, as shown in Table 3, there is also the presence of discriminant validity as the AVE values for one of the two constructions are greater than the square of their correlation estimates, values of diagonal. Therefore, it can be concluded that each measurement item is one-dimensional and represents only its loaded construct but with the exception of two CSR indicators, which is evident since both variables were used to measure CSR compliance and implementation of CSR.

**Table 3:** Discriminant validity.

**Constructs**	**cost_red**	**emp_welb**	**la_resou**	**csr_mgt**	**csr_comp**
cost_red	**0.66**				
emp_welb	0.46	**0.5**			
la_resou	0.03	0.16	**0.5**		
csr_mgt	0.6	0.5	0.16	**0.6**	
csr_comp	0.42	0.3	0.04	0.81	**0.5**

Bold represent the Average Variance Extracted (AVE), of each variable, which is supposed to be higher than the correlation of that variable with other variables.

### Path analysis for the structural equation modeling

**Figure 3:**
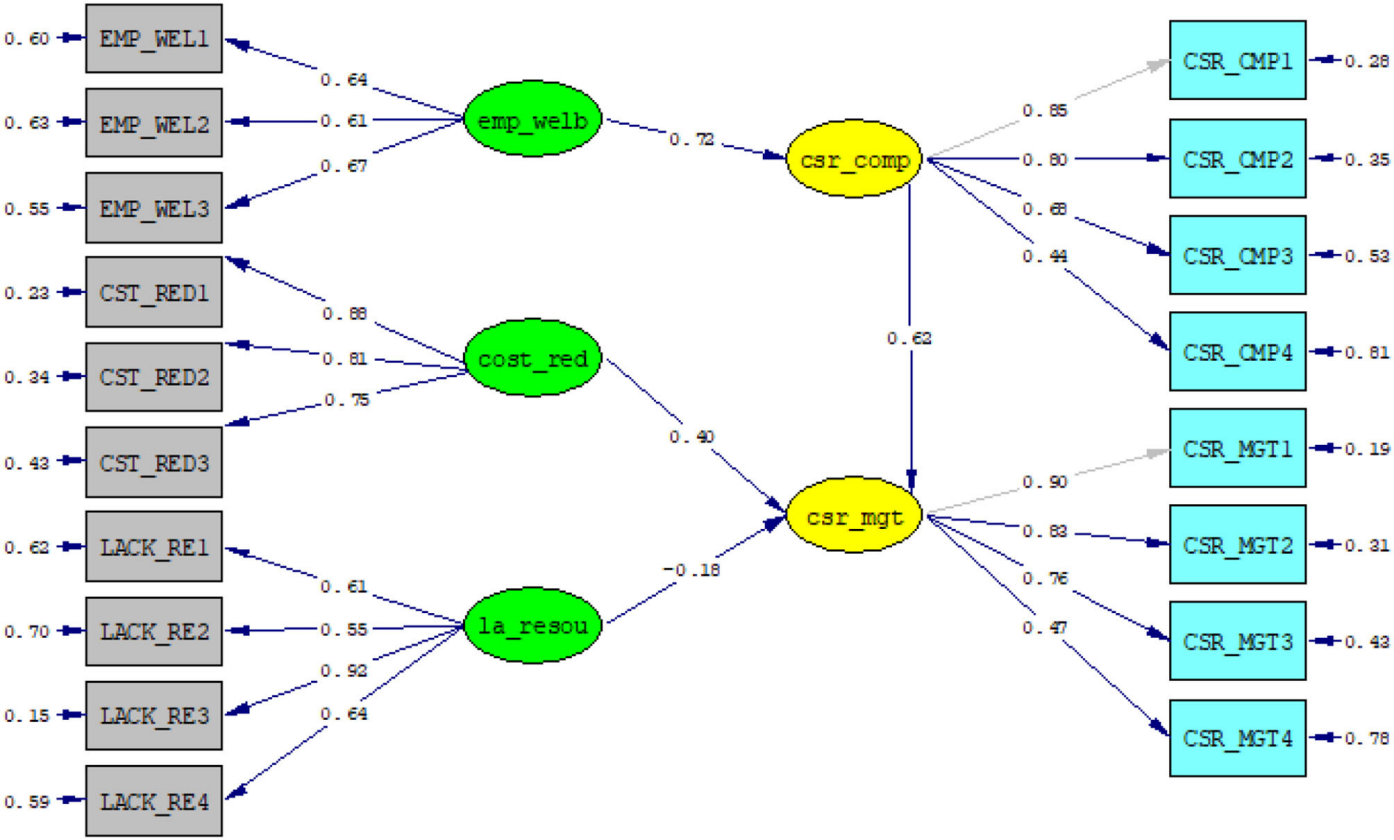
Structural model.

Figure 3 shows a structural model that allows for the researchers to assess the relationship between endogenous variables and exogenous ones. The path, also known as the path coefficients, carries the weights for estimating the significance of the structural relationship and investigating the effects of the exogenous variables on the endogenous ones. Based on the Figure, it can be seen that CSR is positively correlated with motivation for cost reduction and employee wellbeing. In addition, the mediation of CSR compliance is positive; therefore, the H3 is supported.

### Bootstrapping analysis

**Figure 4:**
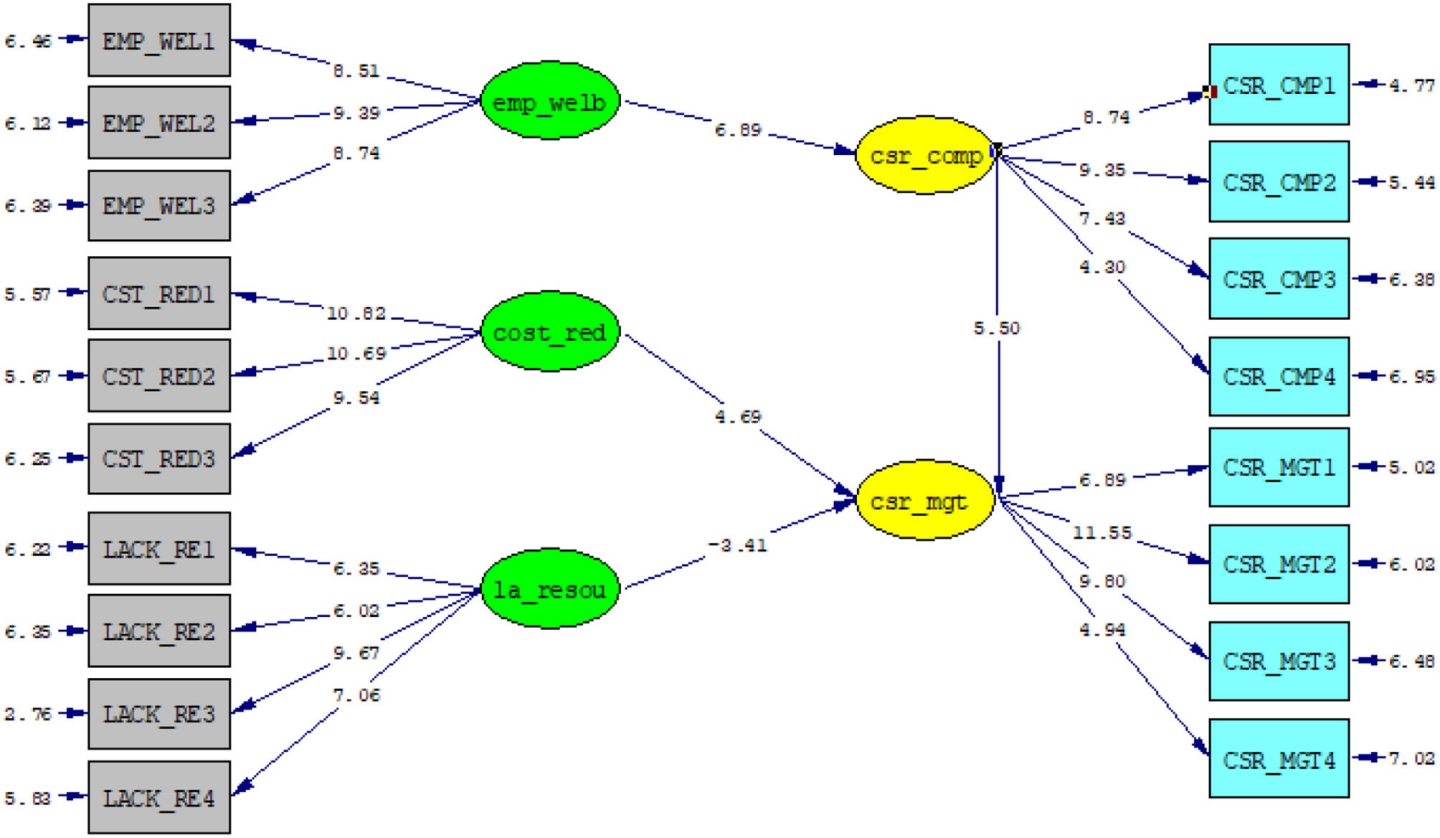
Bootstrapping analysis.

Bootstrapping analysis goes beyond drawing a conclusion based on the statistical inference of distribution. Instead of making assumptions about the statistical inference of the distribution, bootstrapping uses the variability in a model to estimate empirical distribution. Essentially, bootstrapping approximate fit indexes in structural equation modeling (SEM) are of great importance to studies, because they produce tractable analytic distributions. By two-tailed *t*-test with a significance level of 5%, the path coefficient of the model is significant if the T-statistics value is larger than 1.96. A bootstrapping procedure was performed to evaluate the statistical significance of each path coefficient. Between cost reduction and CSR_mgt, T value of 4.69, lack of resource and csr_mgt. Figure 4 illustrates the bootstrapping analysis.

### Mediation analysis

**Figure 5:**
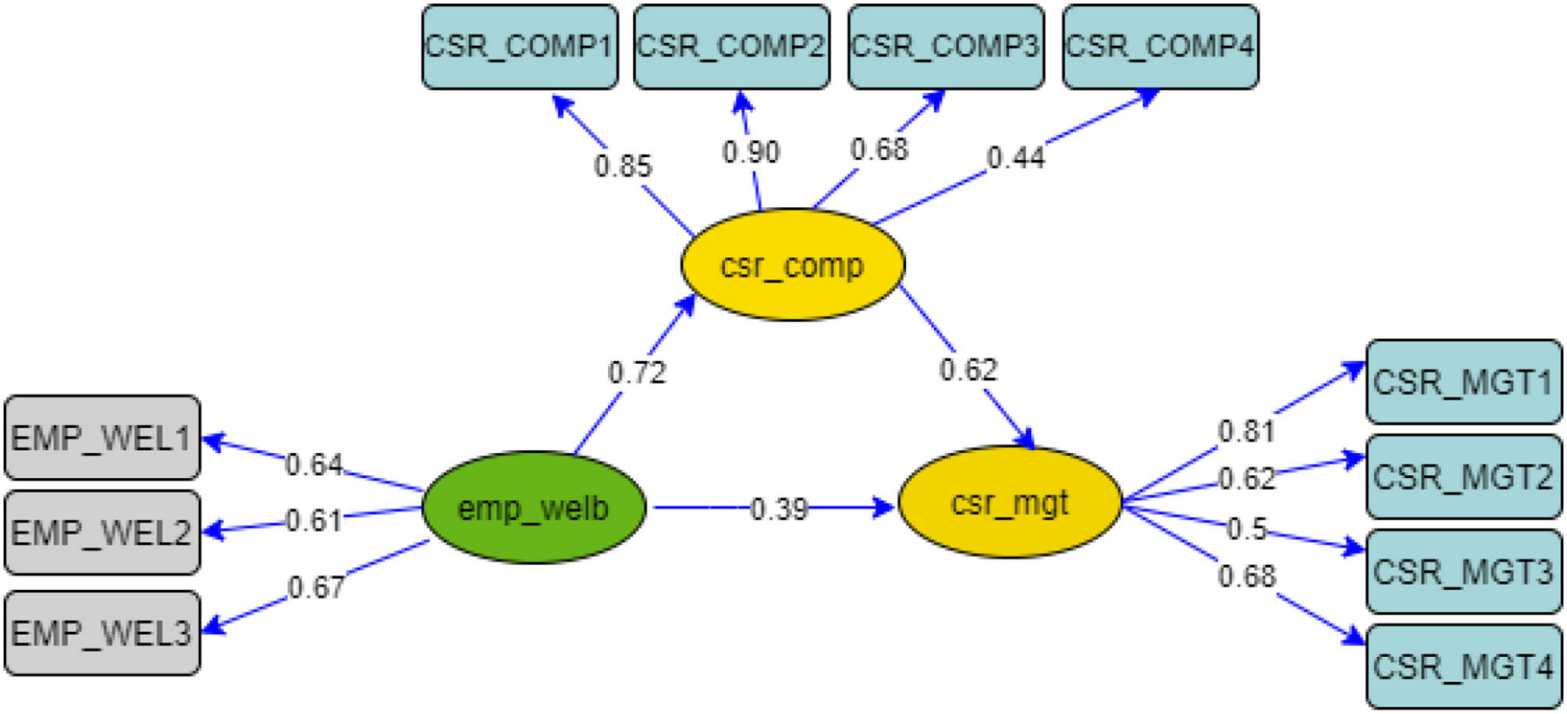
Mediation analysis.

Mediation analysis is defined as the process by which a variable conveys an effect on another through one or more mediating variables. A mediation analysis is conducted is to investigate the casual relationship between the exogenous variable (emp_welb) and the endogenous variable (csr_mgt) by including the third explanatory mediating variable (csr_comp) (Hair et al., 2013). The research model has one mediation effect: the influence of CSR compliance. Figure 5 shows the mediation analysis.

In this mediation, the total effects and indirect effects will be estimated before determining the state of the mediation with respect to the variance accounted for (VAF). Basically, in order to estimate the total effect, summation is carried of direct and indirect effects. They can also be referred to as sums of powers of coefficient matrices. Direct effects are influences that are not mediated by any other variable in the model. Indirect effects are associations of one variable with another mediated by at least one mediating variable. They can also be determined by subtracting direct effects from total effects.

Mediation effect was assessed by variance accounted for (VAF) values of mediated variables to know how much the indirect path is absorbed. According to Hair et al. (2013), the value of VAF helps in determining the mediation effect.

Therefore, the conditions below are mentioned:

i. When VAF lies between 0 < VAF < 0.2, there is no mediation.
ii. When VAF lies between 0.2 < VAF < 0.8, there is partial mediation.
iii. When the VAF value is >0.8, it indicates the presence of full mediation.

The mediation analysis conducted in this investigation makes it possible to estimate the degree of the indirect effect of mediating variable (CSR compliance) between employee wellbeing and CSR implementation, whereby they are considered as the exogenous and endogenous variable for this investigation.

As shown in Table 4, the variance accounted for (VAF) value is 0.533 (53.3%); referring to the mediation condition, this value is satisfying, as it responds to the criteria for partial mediation; therefore, this means that CSR compliance partially mediates the relationship between wellbeing of employee and CSR implementation.

**Table 4:** Mediation analysis VAF.

**Exogenous variable**	**Direct effect**	**indirect effect**	**Total effect**	**VAF RANGE**	**Mediation**
Emp_welb	0.39	0.4464	0.8364	0.533	Partial

Mediating variable: CSR compliance; Endogenous variable: csr_mgt.

### Hypothesis testing

In the results above, the research attempt to model in structural equation in order to determine the influence that the internal factors would have on the effectiveness of CSR implementation. Therefore, we choose to show standardized estimates in the model in order to facilitate the interpretation. First, The R^2^ of the model including the mediator is 90%, which indicates that the internally driven motivation would explain the majority of the variances in CSR implementation. Second, without the mediator, R^2^ is 0.76, which still provides enough information on the variance in CSR implementation. However, this analysis allows to focus on the particularity of this where the relationship between the variables employee wellbeing (emp_welb) and effective CSR implementation (csr_mgt) are mediated by CSR compliance (csr_comp) (β = 0.62). Compliance seems an important indicator in predicting the effectiveness of CSR implementation, because it allows for managers to know more about standards and regulations related to labor. Hence, CSR compliance intervenes in the relationship between employee wellbeing and CSR implementation, and the effect is positive and significant. Then, because of the fact of being motivated in seeking the good of the employees, companies comply with the standards and regulations; this drives companies to compliance (β = 0.72). Meanwhile, for the other two factors, we have chosen to focus on direct relationships that may exist with CSR implementation. Looking at the above figure, it shows that cost reduction (cost_red) influences in a positive way CSR implementation (csr_mgt) by (β = 0.4), which means the motivation to reduce the operating costs in the mining sector seems to be a positive factor that causes mining companies to be efficient in their CSR programs.

The hypothesis on reducing production costs is also accepted in the context of the Congolese mining sector, and the positive relationship between motivation for reducing the operating costs and CSR among mining firms remains acceptable and confirms our observation in the field during the exploratory study where several managers confirmed to install containers to recover waste from e wood, plastics and paper as well as renewal of water and electricity installations in order to reduce their cost. This result also joins the finding of several studies [2]. For instance, the whole literature affirms that firms are motivated by reduction in loads and production costs to implement CSR (Quazi et al., 2001; Melsa, 2006).

Although the structural paths that emanate from the internal factors to CSR implementation are all significant (*p* < 0.05), it is important to note the negative influence of the variable lack of resource on implementation of CSR (β = −18). Managers believe that with enough resources their CSR plans will be achieved. Therefore, implementation of CSR seems to depend on factors specific to the company; lack of resources becomes a barrier to implementation. This study joins previous researchers on the question of lack of resources in the implementation of CSR, which appeared to negatively affect firms insofar as lack of financial resources is often considered as the major obstacle perceived by SME managers (Hillary, 2000; Bowen, 2002; Céspedes-Lorente et al., 2003). Similarly, the managers of mining firms in DRC also expressed the same feeling on this issue.

Thus, integrating green technology is a costly proposition, the economic benefits of which are uncertain and in the long term. For instance, it has been found that large companies have more limited resources and tend to experience fewer related problems when implementing CSR (Fu and Jia, 2012). In addition, they tend to receive more scrutinies from the public for their commitment to CSR. It is also sensitive for large, diversified companies to implement CSR as they can spread their CSR costs over a wider range of business units and services to take advantage of economies of scale. Internally, lack of financial and human resources has often handicapped most firms in the mining sector relative to their CSR strategic approaches. In other word, the lack of financial means and human resources, demanded by the social approach, slows down the motivation of firms to engage in CSR (Table 5).

**Table 5:** Hypotheses summary.

H1: The wellbeing of employees positively influences the effectiveness of CSR implementation	Accepted
H1a: CSR Compliance mediates the relationship between wellbeing of employees and CSR implementation	Accepted
H2: Reducing production costs positively influences the effectiveness of CSR implementation	Accepted
H3: The lack of resources (financial and human) negatively influences the effectiveness of CSR management in the mining sector	Accepted

## Discussion

The above empirical study explains the internally driven motivation toward CSR implementation. The identified motivations all appeared to be associated with implementation of CSR. The structural model shows that cost reduction has a positive impact on CSR, and that wellbeing of employees also motivates managers to engage in CSR by compliance with labor standards in the mining sector. However, lack of resource remains a huge barrier to CSR, as it shows a negative influence on CSR implementation.

Compliance plays an important role in implementing CSR practices at the organizational level by conforming to labor standards and regulations. With firms being motivated to secure the wellbeing of employees, firms ought to comply with labor standards, and with employees being the very key to the firm's strategy, managers strive to ensure the wellbeing of their employees by complying with and engaging in labor laws. Therefore, as it automatically results in firms seeking to enforce the implementation of CSR in an organization to make sure that internally they are meeting the wellbeing of their workers. In the mining sector, for instance, the department of safety and health is very essential in making sure workers are in good condition.

While firms are motivated to reduce their production cost, most strategies are CSR practices that firms put in place and allow mining firms to effectively manage their production cost. Therefore, this study joins the studies of Sprinkle and Maines, who earlier suggested that cost reduction would motivate firms to engage in CSR. Additionally, this finding reveals that the top managers in the mining sector are aware that implementing CSR would allow for their firms to get certain benefits. Firms aim to lower their usage of energy and fuel and save money by having lower bills. Firms implement it as a differentiation strategy by becoming greener through their service following with their CSR activities.

The findings of this study concur with existing research studies that have argued on the significance of resources in implementing CSR; this study has revealed, with evidence from the mining sector, that lack of resource was negatively correlated with CSR implementation. Lack of resources can be manifested in various forms and can be a barrier in implementing CSR. Therefore, this is a remainder to most mining firms to start allocating significant budgets for implementing CSR. For instance, firms with weak financial capabilities, firms that do not have enough human capital, and firms that struggle with knowledge and expertise might not be effective in their CSR strategy. Similarly, Lam and Lim (2016), in their research studies on effective implementation of CSR, argued that a large number of resources were demanded.

## Conclusion

This research has established a framework that explains the implementation of CSR through the internally driven motivation of employee wellbeing and cost reduction in the mining sector of the DRC. The internal drivers are all significant, whereas lack of resource is negatively impacting the implementation. This study adds to the limited literature on CSR in the mining sector, to rare studies that explore the motivations of implementing CSR in the mining sector, and to the research on CSR at micro level by considering that the research sought the perceptions of individuals or managers.

Subsequently, the Congolese government should regularly monitor the progress of the CSR process through its specialized bodies and clearly formulate its positions that will serve as guidelines for mining companies. It would be realistic to assume that a structure combining voluntary and regulatory standards is the most effective path for CSR. Therefore, policymakers can, on the other hand, come up with policies and provide support mining firms in their efforts to integrate CSR in their day-to-day operations. This research suggests to policymakers and institutional bodies in DRC to intensify the regulations and encourage voluntary standards in line with mining workers, as firms are motivated to improve the wellbeing of their employee through these standards. This will allow for firms to commit to CSR and comply with the regulations. However, establishment of self-regulatory initiatives in the Congolese mining industry should therefore be seen in the context of a more in-depth social and environmental review of the mining firms.

Thus, it is important to underscore that there remains some uncertainty concerning the quality of the measures of the variables, in particular those measured by scales constructed around subsequent empirical studies and exploratory surveys. Likewise, certain measurement scales were readjusted and adapted for the need and context of the research, needed to be reused, or even improved, in similar contexts, in order to improve their measurement qualities. Another limitation is the use of the questionnaire as a mode of data collection. This limitation lies in the fact that the answers do not really correspond to reality. This difficulty is in fact due to the gap between what is declared and what is practiced. Despite the efforts made to reduce this lag, this limit should be taken into account in the sense of using the results with caution. Lastly, as this study is mainly about identifying the motivations to a better implementation of CSR, therefore the solutions to improvement would have been discussed as well through management strategies. However, this research just covered a small portion, a call for future research to focus on identifying barriers to CSR in the mining sector. Moreover, this research encourages identifying various factors of CSR implementation, and future research should consider the individual characteristics of managers as mediators, and the research can be extended to other sectors. As a continuation of this study, this issue could be extended to other sectors or other countries that exploit the same cultural environment (sub-Saharan region), and to all sectors where CSR is highlighted, and longitudinal analysis should complement the conclusions of this research in order to confirm the causal relationships.

## Data availability statement

The original contributions presented in the study are included in the article/Supplementary material, further inquiries can be directed to the corresponding author/s.

## Author contributions

HS performed research supervision and funding acquisition. GB analyzed and interpreted the data and wrote the article. Both authors contributed to the article and approved the submitted version.
