# Supplementary material for: Employee wellbeing and cost reduction drivers of corporate social responsibility: Evidence from Congolese mining sector
Source: Front Psychol. 2023 Feb 6;13:850283. doi: 10.3389/fpsyg.2022.850283 (PMC9940710; doi:10.3389/fpsyg.2022.850283)
Supplement: Supplementary file 1 [file Data_Sheet_1.pdf]

Appendix

Top managers were asked to indicate the extent to which they agree with the statements below, using a scale of Likert of 5 points, ranging from Strongly Disagree to Strongly Agree.

Appendix A

**Cost reduction**	**1**	**2**	**3**	**4**	**5**
The company is motivated by reducing the consumption of energies and waste discharges.					
The company is motivated by the reduction of risks operational costs.					
The company is motivated by increasing productivity and profitability.					
**Employee well being**	1	2	3	4	5
The company is motivated by improving the measures of protection of the health of employees at work (chemical gases, steam, dust, fumes, odors, nuisances etc.)					
The company is motivated by improving the environment, safety and hygiene at production sites.					
The company is motivated by improving working hours, in order to reconcile the work and the private life of the employees					
**Lack of resource**	**1**	**2**	**3**	**4**	**5**
The company is hampered by the lack of financial means					
The company is hampered by the lack of competent employees					
The company is hampered by the lack of competent employees					
The company is held back by the lack of interest in this issue					

Appendix B

CSR implementation	1	2	3	4	5
The company has appointed a committee that oversees CSR practices					
This firm has got a well written CSR policy					
This firm has been awarded for implementing CSR effectively in our operations , received CSR type certificates or award					
This firm implements and promotes CSR standards					
**CSR Compliance**	**1**	**2**	**3**	**4**	**5**
In this firm all permanent employees have a written labor contract					
the firm has a local trade union					
the firm pays social insurance					
The firm pays health insurance to employees					
